# Equivalent Air Spring Suspension Model for Quarter-Passive Model of Passenger Vehicles

**DOI:** 10.1155/2015/974020

**Published:** 2015-05-31

**Authors:** Haider J. Abid, Jie Chen, Ameen A. Nassar

**Affiliations:** ^1^Department of Mechanical Engineering, Thi-Qar University, Nasiriyah, Iraq; ^2^Department of Mechanical, Aerospace and Civil Engineering, Brunel University, Uxbridge UB8 3PH, UK; ^3^Department of Mechanical Engineering, Basrah University, Basrah, Iraq

## Abstract

This paper investigates the GENSIS air spring suspension system equivalence to a passive suspension system. The SIMULINK simulation together with the OptiY optimization is used to obtain the air spring suspension model equivalent to passive suspension system, where the car body response difference from both systems with the same road profile inputs is used as the objective function for optimization (OptiY program). The parameters of air spring system such as initial pressure, volume of bag, length of surge pipe, diameter of surge pipe, and volume of reservoir are obtained from optimization. The simulation results show that the air spring suspension equivalent system can produce responses very close to the passive suspension system.

## 1. Introduction

Suspension system design is a challenging task for the automobile designers in view of multiple control parameters, complex objectives (often conflicting), and stochastic disturbances. The problems stem from the wide range of operating conditions created by varying road conditions, vehicle speed, and load [1]; in general, the road handling and safety rules request harsh suspensions, while the passengers comfort feeling requires a soft damping. There are three types of suspension system: passive systems, semiactive systems, and active systems. Each of the type of suspension has different advantages and disadvantages. Passive suspension systems are subject to various tradeoffs when they are excited across a large frequency bandwidth [2], the disadvantage of a semiactive suspension where the system can be controlled only in one direction: opposite to the velocity of the damper extension [3]. The active vibration control has the disadvantages of complexity and high-energy consumption. The air spring suspension systems can be used to overcome these difficulties. The air spring system is well known for its low transmissibility coefficients and its ability to vary load capacities with only the change of the gas pressure within the springs. Air springs can be used for a mechatronic approach in suspension design because of their ability to provide a controlled variable spring rate and they offer simple and inexpensive automatic leveling.

One of the advantages of air springs is that the energy-storage capacity of air is far greater per unit weight than that of mechanical spring material, such as steel. Because of the efficient potential energy storage of springs of this type, their use in a vibration-isolation system can result in a natural frequency for the system which is almost 10 times lower than for a system employing vibration isolators made from steel springs [4]. This leads to the applications where low frequency vibration isolation is necessary [5]. Air springs have not only lower resonance frequencies but also smaller over-all length than mechanical springs with equivalent characteristics [6]. The ability to change the load carrying capacity simply by changing the air pressure rather than changing out the air spring is a major advantage that air springs have over steel springs. The air spring is mainly used in commercial vehicles, but lately it is also used in higher classes of passenger vehicles.

As discussed above, the air spring suspension has a number of advantages in real application. However the design suspension system with air springs has been studied extensively. On the contrary, the design and analysis of the passive suspension system are fully established. Therefore, the air spring suspension system design problem can be converted to a model which produces suspension performance the same as a passive suspension system when using it without active controller or it can produce more efficient suspension system when use it with active controller. It is possible to convert a passive suspension system to an air spring suspension system and vice versa [7–12]. The equivalence means that both systems have the similar suspension response for the same road profile inputs.

There have been some researches on the use of air spring for suspension systems. For example, Toyofuku et al. showed that the auxiliary chamber has a smaller effect on the system [13] in high frequencies. Bhave studied the effects of pitch interconnection on vehicle performance and ride comfort by using completed parametric modeling [14]. Crolla and Ramsbottom made some improvements in performance for roll behavior of vehicle when he used electronically controlled pneumatic suspension [15]. Xiao et al. developed force-deflection relationship based on experimental data of nonlinear air spring model, where the sliding mode controller for quarter car model is used for the half car model of a vehicle suspension [16]. The finite element method was used to analyse the air spring stiffness by Wu et al. [17]. Moreover, the paper [17] compared the stiffness of equivalent of air spring model with stiffness of original model and discussed the relationship between inherent frequency and initial pressure, between air spring stiffness and auxiliary chamber, between air spring stiffness and different initial pressure. The pressure of air supplied to the electropneumatic pressure regulator was controlled by regulating the voltage provider by Bhandari and Subramanian [18]. Bruni et al. investigated the dynamic characteristics of an air spring suspension with control [19].

The main objective of this study is to obtain an air spring suspension system which can replicate the passive suspension system in terms of the suspension performance. In this research, an air spring suspension system is to be found which can produce the performance better than VAMPIC model [20] under the simulated quasistatic stiffness of the suspension. In the following part of the paper, the mathematical models for both air spring and passive suspension are presented in Section 2 and the optimization method is used to find air spring suspension model parameters in Section 3.

## 2. Air Suspension System Mathematical Model and Its Passive Suspension System Equivalence

### 2.1. Air Suspension System

**Figure 1:**
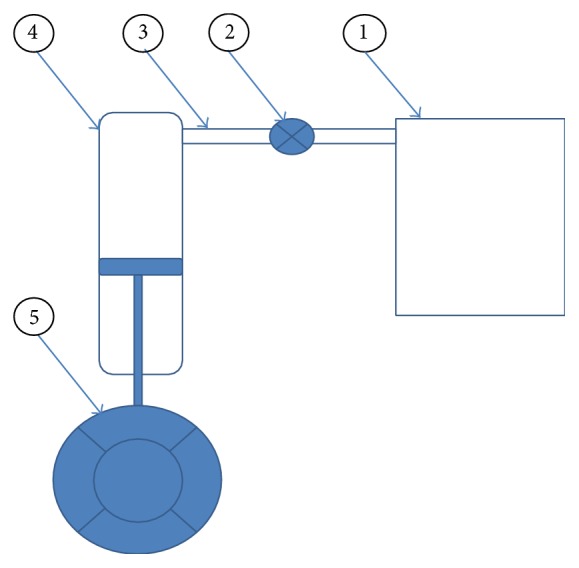
Air spring suspension system: (1) reservoir, (2) controlled valve, (3) surge pipe, (4) air bag, and (5) tire.

The basis for mathematical models of air springs is to measure its mechanical properties. The mechanical behavior of air springs is often very complicated. The behavior is mainly based on fluid dynamic and thermodynamic mechanisms, where important quantities in such mechanisms are pressure, volume, temperature, mass flow rate, density, and energy of the air as well as shape of the air volume. For most air springs, these quantities should be expressed for both the air spring itself and its reservoir volume, as shown in Figure 1.

### 2.2. Air Spring Modeling

There are many different kinds of air spring models, such as a simple model for vertical air spring dynamics (Nishimura [6], VAMPIRE [21], SIMPAC [22], and GENSYS [23, 24]). The air spring system as illustrated in Figure 1 consists of an air bag connected to a reservoir by a pipeline system and a controlled valve. The system's stiffness can be changed. The modeling of an air spring, presented here, does not take in consideration the leveling system because these changes are very slow. The mathematical models incorporate the stiffness and the damping characteristics of the air spring. Under the vibrations, the behavior of the compressed air within the air spring system is polytropic. The minimal stiffness is reached when there is an isothermal change of the gas state (for frequencies *f* < 0.1 Hz), and the maximal stiffness is associated with adiabatic state change (for frequencies *f* > 3 Hz). The analysis of the vehicle vertical dynamics shows a special interest around the frequency domain from 0 to 20 Hz [6].

In order to consider the change in the gas state in the two volumes, an approximation has been introduced by implementing a mechanical barrier (fictive piston) in the pipeline. The mechanical barrier is considered to be with neglected mass and equivalent fluid mass that is moving through the pipeline is added to the barrier [5]. This approximation is justified because small amount of fluid oscillates between the two volumes.

**Figure 2:**
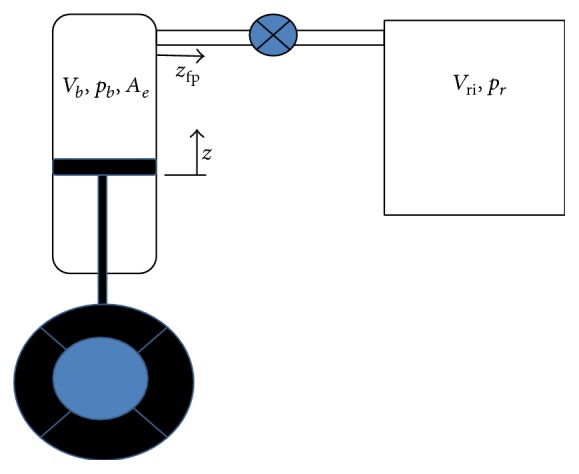
Modeling of air suspension spring.

The following analysis follows the method of calculation in [25] with the simplified air spring system shown in Figure 2. After a defection the new air bag volume and new reservoir volume with polytropic process, we get

(1) Vb=Vbi−zAe+zfpAsVrVri−zfpAs,

where *z* is the deflection of air bag. *z*_fp_ is the the displacement of air in surge pipe. *A*_*e*_ is the the effective area of air bag. *A*_*s*_ is the the cross section area of the pipeline. *V*_bi_ is the the initial volume of air bag. *V*_ri_ is the the initial volume of reservoir.

**Figure 3:**
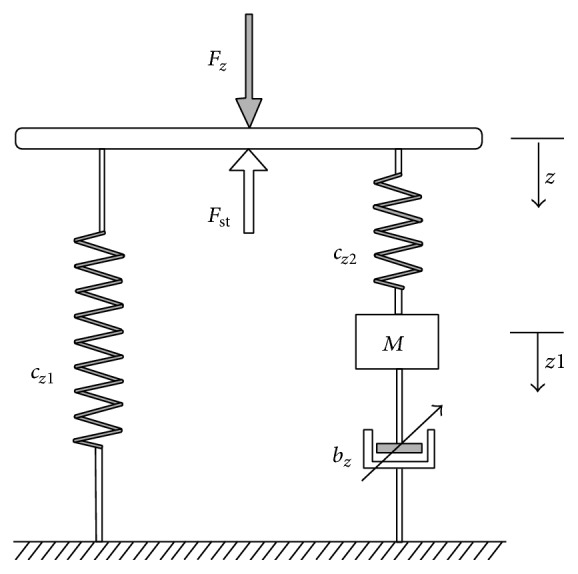
The mechanical model of air suspension system [6].

The GENSYS model of the air suspension system as shown in Figure 3 has polytrope gas state change [6]. For this mode, the static load and the stiffness constants *C*_*z*1_, *C*_*z*2_, *M*, and *b*_*z*_ can be identified as

(2) Cz1piAe2nVbi+VriCz2piAe2nVbi+Vri⁡VriVbi=Cz2VriVbiMlsAsρAeAsVriVbi+Vri2.

The model parameter *b*_*z*_ is related to the velocity over the damper (*b*_*z*_) and not related to the velocity in the surge pipe. According to [23], the vertical viscous force is written as

(3) $$\begin{array}{c} F_{vz}=C_{z2}\left(\;z-w_{s}\;\right)=b_{z}{\left|\;{\dot{w}}_{s}\;\right|}^{\beta }\mathrm{sign}\left(\;{\dot{w}}_{s}\;\right)+M{\ddot{w}}_{s}. \end{array}$$

The above expression can be rewritten as

(4) $$\begin{array}{c} M{\ddot{w}}_{s}=C_{z2}\left(\;z-w_{s}\;\right)-b_{z}{\left|\;{\dot{w}}_{s}\;\right|}^{\beta }\mathrm{sign}\left(\;{\dot{w}}_{s}\;\right). \end{array}$$

The relationship between the nonlinear damping *C*_*zβ*_ and *C*_*s*_ is

(5) $$\begin{array}{c} b_{z}=b_{s}k_{wz}^{1+\beta }=b_{s}{\left(\;\frac{A_{e}}{A_{s}}\frac{V_{\mathrm{ri}}}{V_{\text{bi}}+V_{\mathrm{ri}}}\;\right)}^{1+\beta }. \end{array}$$

The value of *b*_*s*_ can be calculated from the air damping occur in all system. The total pressure loss in a typical surge pipe occurs due to the loss of energy as major and minor fluid losses. The source of these energy losses is the abrupt enlargements and contractions where the air bag and reservoir joins the surge pipe, pipe friction, the number of pipe bends, and the loss in controlled valve (which is used with future study and it is not included with the determination of loss in this study). This gives that the total loss coefficient contains the following parts:

(6) bs12·ρi·bst·Asbst=bfr+ben+bc+bb,

where *b*_st_ is the is the total loss coefficient, *b*_fr_ is the loss coefficient due to friction, *b*_en_ is the loss coefficient due to enlargement, *b*_*c*_ is the loss coefficient due to contraction, and *b*_*b*_ is the loss coefficient due to bends in the pipe.

### 2.3. Mathematical Model of Passive Suspension System

**Figure 4:**
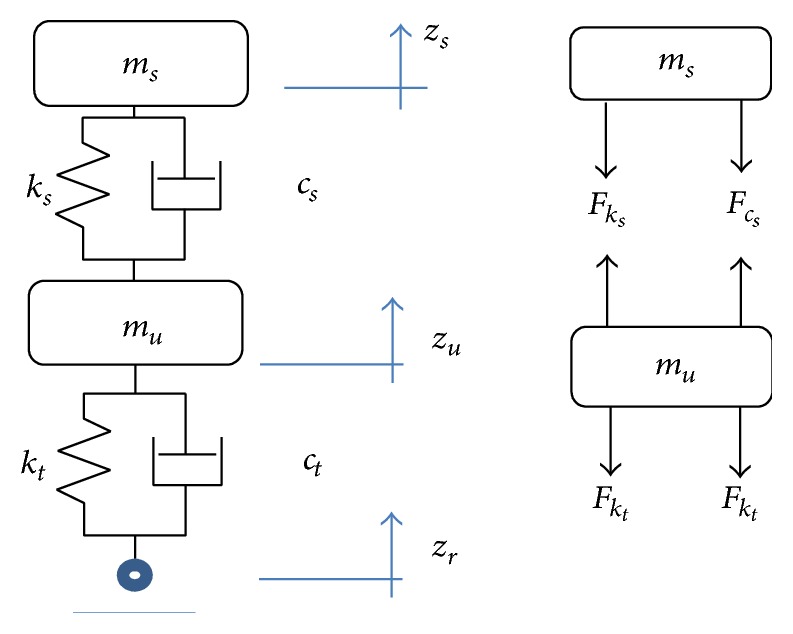
Quarter-car model and relevant free body diagram.

The quarter car model of passive suspension system of vehicle is shown in Figure 4. It can be a simplified model with lumped masses and their relevant elements. In this study, the fundamental parameters of a suspension system can be defined by this model.

Nonlinear equations of the sprung mass and unsprung mass motions can be derived in two parts as follows [26].

Sprung mass equation:

(7) msz¨=−kszs−zu−ζkszs−zu3−cszs˙−zu˙−ζcszs˙−zu˙2sgn⁡zs˙−zu˙.

Unsprung mass equation:

(8) muzu¨=kszs−zu+ζkszs−zu3+cszs˙−zu˙+ζcszs˙−zu˙2sgn⁡zs˙−zu˙−ktzu−zr−ζktzu−zr3−ctzu˙−z˙r−ζctzu˙−zr˙2sgn⁡zu˙−zr˙,

where *F*_*k*_*s*__ is the nonlinear force of coil springs (N). *F*_*c*_*s*__ is the nonlinear forces of damper (N). *F*_*k*_*t*__ is the the spring force of tire (N). *F*_*c*_*t*__ is the the damping force of tire (N). *m*_*s*_ is the mass of vehicle's body (kg). *m*_*u*_ is the mass of wheel (kg). *k*_*s*_ is the stiffness of spring (N/m). *k*_*t*_ is the stiffness of tire (N/m). *c*_*s*_ is the damping coefficient of damper (N·s/m). *c*_*t*_ is the damping coefficient of tire (N·s /m). *z*_*s*_ is the displacement of vehicle's body (m). *z*_*u*_ is the displacement of wheel (m). *z*_*r*_ is the displacement of road profile (m). *ζ* is the empirical parameter

## 3. Obtain Equivalent Air Spring Suspension Model Using Optimization

The idea of equivalent model is to find an air spring suspension system configuration which produces the same suspension performance (displacement) as a passive suspension system count part with the same road profile inputs (if the passive mode is selected that means without controller). This is achieved by finding air spring system model parameters by minimizing the performance difference.

**Figure 5:**
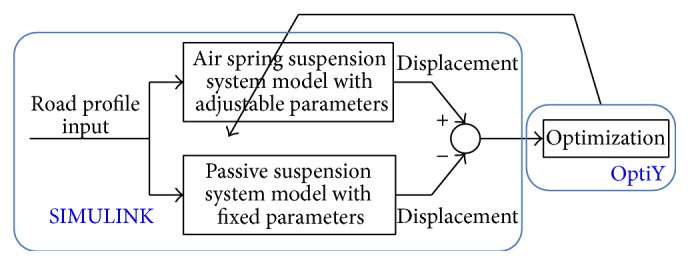
Finding equivalent model using SIMULINK simulation and OptiY optimization.

The process of obtaining the equivalent model is illustrated by Figure 5 where the response of both air spring suspension and passive suspension models are simulated using SIMULINK. The OptiY optimization program is used to adjust air suspension model parameters so that the response difference is minimized. The air suspension model parameters to be found are initial pressure in system, volume of bag, length of surge pipe, diameter of surge pipe, and volume of reservoir. The optimization is a constraint optimization problem where the design parameter constraints are given in Table 1.

The parameter values of the quarter passive vehicle model used in the simulation are taken from [27]. The road profile used in this study is sine, square, and saw tooth waves with amplitude 10 cm and frequency of 1 Hz. The optimization technique is implemented with 3000 attempts and the SIMULINK simulate the systems with 20 seconds period. The optimal parameters found are presented in Table 2.

**Figure 6:**
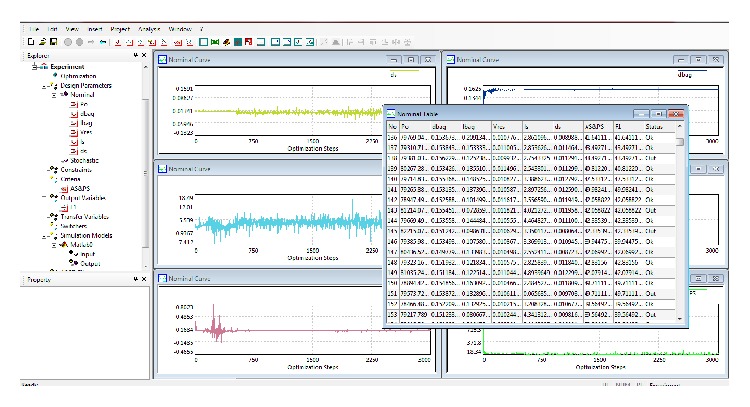
The results of OptiY software with MATLAB/Simulink.

**Figure 7:**
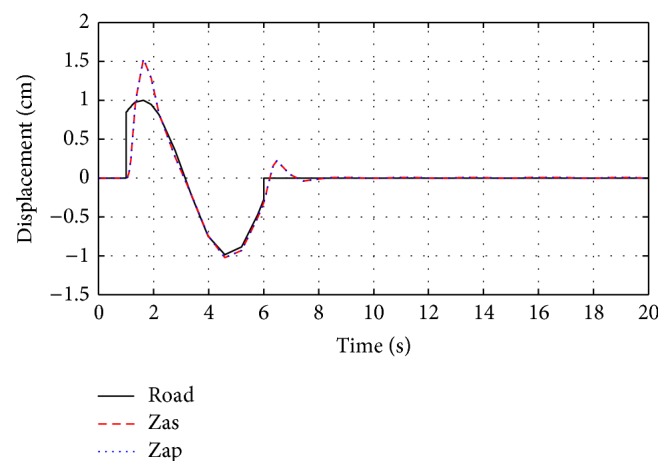
The response of optimization technique with sine wave road profile.

**Figure 8 fig8:**
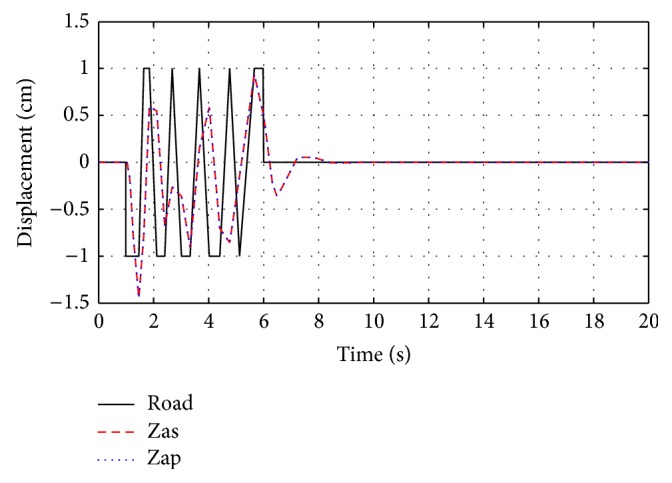
The response of optimization technique with square wave road profile.

**Figure 9:**
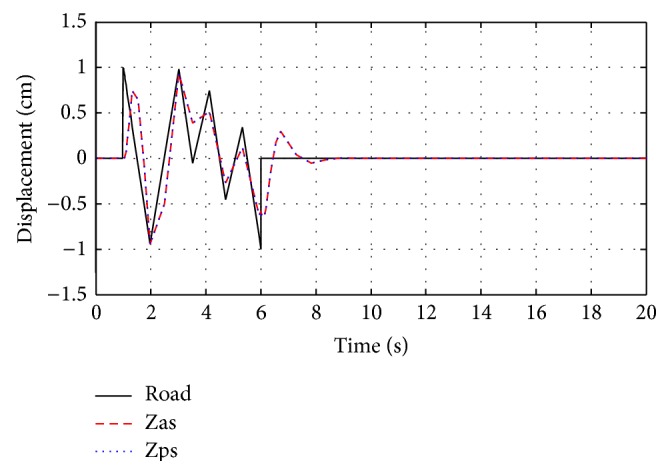
The response of optimization technique with saw tooth wave road profile.

The system responses for passive and optimized air spring suspension systems are presented in Figure 6. The response of equivalent air spring suspension system with sine wave road profile, square road profile, and random road profile are shown in Figures 7 [Fig fig8]–9.

## 4. Conclusion

The optimization technique available in OptiY with SIMULINK simulation was successfully used in this paper to find the equivalent air spring suspension model with the optimized parameters. The results are shown that the equivalent model can produce the suspension response similar to the passive suspension system. In future studies, the model can be used in active air suspension system design for better handling and stability properties. The nature of low resonance frequency in air spring can be exploited by frequency domain controller design methods such as H∞ methods [28].

## Figures and Tables

**Table 1:** The lower bound and the upper bound of the constraint parameters.

Parameter	Lower bound	Upper bound	Unit
Initial pressure (in bag and reservoir)	100000	700000	kPa
Bag diameter	0.05	0.2	m
Bag height	0.1	0.75	m
Reservoir volume	0.01	0.3	m^3^
Length of surge pipe	1	5	m
Diameter of surge pipe	0.003	0.025	m

**Table 2:** The numerical values of the passive suspension system.

Notations	Description	Values	Units
*K*1, *K*2	Front-left and front-right suspension stuffiness, respectively	19960	N/m
*K*3, *K*4	Rear-left and Rear-right suspension stuffiness, respectively	17500	N/m
*k*1–*k*4	Front-left and front-right and rear-right and rear-left tire stuffiness, respectively	175500	N/m
*C*1, *C*2	Front-left and front-right suspension damping, respectively	1290	N·s/m
*C*3, *C*4	Rear-left and rear-right suspension damping, respectively	1690	N·s/m
*c*1–*c*4	Front-left and front-right and rear-right and rear-left tire damping, respectively	14.6	N·s/m
*M*	Sprung mass	1460	Kg
*m*1-*m*2	Front-left and front-right tire mass respectively	40	Kg
*m*3-*m*4	Rear-left and Rear-right tire mass respectively	35.5	Kg
*Jx*	Moment of inertia *x*-direction	460	Kg·m^2^
*Jy*	Moment of inertia *y*-direction	2460	Kg·m^2^
*l*_1_	Distance between the center of gravity of vehicle body and front axle	1.011	m
*l*_2_	Distance between the center of gravity of vehicle body and rear axle	1.803	m
*b*	Width of track	1.51	m
